# A Village Committee

**Published:** 1918-07-06

**Authors:** 


### A Village Committee.

THE columns of THE HOSPITAL have always been open to record interesting social events on behalf of public causes. The letter which we now give contains an account of an annual parade, which resulted in a net sum of £180 being handed over to the Leicester Royal Infirmary. It is interesting not only for the details of the harmonious working of a village committee for many weeks, but because it breathes the spirit of gratitude. Our correspondent writes: "I thought it might interest you to hear what a great day we had last Monday week (Whit Monday). In the first place, the weather was ideal, and there is no doubt that our success is largely due to that fact. There were many hundreds of people from neighbouring villages, and it was a good sight to see the streets so full. There were so many passengers for the 12.20 train that they stopped issuing tickets, and a large number of would-be visitors were left on the platform. The street collection was easily a record.

"We had 3,200 persons pay for admission to the park after the parade, the best we have had to date, the vehicles, cycles, perambulators, costumes, etc., being very numerous and excellent. Mrs. Jones and Mrs. Lockton arranged a rummage sale on the grounds, and handed over as the result £16 12s. 6d. Mrs. Underwood has been at work for several weeks making articles, and she handed to me £11. Miss Emily Betts also made articles, and realised £5 12s. A discharged soldier, using the talent picked up in hospitals, made links of beads, and we gained through his work £4.

"Messrs. S. Widdowson and H. Dring organised a skittle competition for the field. They bought a pig as a prize, giving 30s. for a £5 pig; they begged the 30s., and made 10 guineas from the competition. Mr. T. Ford decided to dress up as a golliwog, and during the day sold small golliwogs at 2d. each, and raised £2 1s. 4d. He made the golliwogs himself during the week before Whitsuntide. There are a host of other good friends who helped us, but it would take me all night to tell you about them. I am just mentioning a few cases to let you see how the people are eager to assist us. There was a time when a good portion of the people were prejudiced against us, but now they simply tumble over one another in their eagerness to help. I believe they are thankful for what the institution has done, and is still doing, and they work like this with thankful hearts. Well, I trust we have not reached the limit yet; at least we will try and beat it next year."

The sentiment contained in the closing paragraph indicates the progressive spirit of the promoters, and we shall hope to record at least £200 next year.

